# Molecular Mechanisms of Stress-Induced Increases in Fear Memory Consolidation within the Amygdala

**DOI:** 10.3389/fnbeh.2016.00191

**Published:** 2016-10-21

**Authors:** Antonio V. Aubry, Peter A. Serrano, Nesha S. Burghardt

**Affiliations:** ^1^Department of Psychology, Hunter College, The City University of New YorkNew York, NY, USA; ^2^The Graduate Center, The City University of New YorkNew York, NY, USA

**Keywords:** GluA2, glucocorticoids, norepinephrine, PTSD, stress, fear conditioning, basolateral amygdala, memory consolidation

## Abstract

Stress can significantly impact brain function and increase the risk for developing various psychiatric disorders. Many of the brain regions that are implicated in psychiatric disorders and are vulnerable to the effects of stress are also involved in mediating emotional learning. Emotional learning has been a subject of intense investigation for the past 30 years, with the vast majority of studies focusing on the amygdala and its role in associative fear learning. However, the mechanisms by which stress affects the amygdala and amygdala-dependent fear memories remain unclear. Here we review the literature on the enhancing effects of acute and chronic stress on the acquisition and/or consolidation of a fear memory, as measured by auditory Pavlovian fear conditioning, and discuss potential mechanisms by which these changes occur in the amygdala. We hypothesize that stress-mediated activation of glucocorticoid receptors (GR) and norepinephrine release within the amygdala leads to the mobilization of α-amino-3-hydroxy-5-methyl-4-isoxazolepropionic acid (AMPA) receptors to the synapse, which underlies stress-induced increases in fear memory. We discuss the implications of this hypothesis for evaluating the effects of stress on extinction and for developing treatments for anxiety disorders. Understanding how stress-induced changes in glucocorticoid and norepinephrine signaling might converge to affect emotional learning by increasing the trafficking of AMPA receptors and enhancing amygdala excitability is a promising area for future research.

## Introduction

Stress is defined as any threat or perceived threat that disturbs an organism’s ability to maintain homeostasis. Although activation of the stress response is initially adaptive, long-term exposure to stress poses a significant risk for the development of numerous psychiatric disorders (Jacobs and Nadel, 1985; Breslau et al., 1999; Kendler et al., 1999; Risch et al., 2009; Briere et al., 2016). Many of the brain regions that are implicated in psychiatric disorders and are vulnerable to the effects of stress are also involved in mediating emotional learning, alterations of which have been suggested to contribute to the onset and maintenance of these disorders (Rothbaum and Davis, 2003). Emotional learning has been a subject of intense investigation for the past 30 years, with the vast majority of studies focusing on the amygdala and its role in associative fear learning. While this has led to identification of many of the underlying mechanisms, it remains unclear how stress affects these mechanisms. A greater understanding of this process is of considerable clinical relevance, particularly for pharmacological interventions that are provided in conjunction with learning-based treatments (Bowers and Ressler, 2015). Here we briefly review the literature on the cellular and molecular mechanisms mediating associative learning, as measured by Pavlovian fear conditioning. We then summarize the effects of acute and chronic stress on the acquisition and consolidation of a fear memory and discuss potential mechanisms by which these changes occur in the amygdala. All the studies that we review used auditory Pavlovian fear conditioning, unless otherwise specified. We hypothesize that stress-mediated activation of the glucocorticoid receptor (GR) and norepinephrine release within the amygdala lead to mobilization of α-amino-3-hydroxy-5-methyl-4-isoxazolepropionic acid (AMPA) receptors to the synapse, which underlies stress-induced increases in fear memory. We discuss the implications of this hypothesis for evaluating the effects of stress on extinction and for developing treatments for anxiety disorders characterized by aberrant fear learning.

## Pavlovian Fear Conditioning as a Model of Associative Learning

Pavlovian fear conditioning has been used extensively to study the cellular and molecular mechanisms of associative learning. In Pavlovian fear conditioning, a neutral stimulus, such as a tone (conditioned stimulus, CS) is paired with an aversive stimulus, such as a foot shock (unconditioned stimulus, US). After the animal learns to associate the CS with the US, presentation of the tone alone elicits defensive responses that are characteristic of fear, such as freezing (LeDoux et al., 1988; Fendt and Fanselow, 1999). This process of fear conditioning involves distinct stages of memory. Acquisition refers to the initial learning of the CS-US association, and is followed by consolidation, which is the period during which the memory is thought to become stable. Long-term memory is typically tested the next day when the CS is presented in the absence of the US, leading to retrieval of the fear memory. Interestingly, retrieval destabilizes the memory and a process known as reconsolidation is required to put it back into long-term storage. When retrieval involves multiple presentations of the CS alone, the CS gradually loses the ability to evoke a conditioned response, demonstrating acquisition of extinction. This form of learning also requires consolidation so that the extinction memory can be retrieved at a later time point.

Numerous studies have established a functional role for specific subnuclei in the amygdala in mediating fear conditioning. These subnuclei include the lateral nucleus (LA), which is the main input nucleus and a key site of plasticity underlying associative learning (Rogan et al., 1997; Fendt and Fanselow, 1999; Rodrigues et al., 2004), the basal nucleus (BA), and the central (CeA) nucleus, which is the main output nucleus of the amygdala. During acquisition, the CS causes glutamate release from pre-synaptic terminals where it binds to AMPA and N-methyl-D-aspartate (NMDA) receptors located on principal cells in the LA. These same cells in the LA are depolarized by somatosensory inputs activated by the foot shock (US; Romanski et al., 1993). Strong depolarization removes the Mg^+^ block from NMDA receptors, allowing for calcium entry (Mayer et al., 1984). Calcium, in turn, activates the Ca^2^/calmodulin dependent protein kinase II (CAMKII), which modulates AMPA receptors by increasing their conductance (Barria et al., 1997a,b) and by increasing their trafficking to the synapse (Krapivinsky et al., 2004). More specifically, CAMKII increases the conductance of GluA1-containing AMPA receptors via phosphorylation mechanisms (Barria et al., 1997a,b) and mobilizes GluA1 to the synapse (Krapivinsky et al., 2004). During the acquisition of a conditioned fear memory, GluA1 is driven into the synapses of dendritic spines of principal neurons in the LA (Nedelescu et al., 2010), a process that is necessary for fear learning (Rumpel et al., 2005). For the memory to be consolidated, activation of protein kinases, such as protein kinase A and mitogen-activated protein kinase, trigger gene transcription and the translation of new proteins (Schafe and LeDoux, 2000; Schafe et al., 2000; Alberini, 2009). Activation of protein kinase A in particular has been shown to increase the synthesis of GluA1 and GluA2-containing AMPA receptors (Nayak et al., 1998). During consolidation, GluA2 subunits replace the newly inserted GluA1 subunits, thereby stabilizing the memory for later retrieval (Joels and Lamprecht, 2010; Hong et al., 2013). Maintenance of GluA2 in the synaptic membrane has been shown to be dependent upon interactions with protein kinase M zeta (PKMζ), which together form a complex that underlies the long-term persistence of conditioned fear memories in the LA (Migues et al., 2010).

## Effects of Acute Stress on Fear Memory: Potential Mechanisms in the BLA

Acute stress rapidly activates the autonomic nervous system, which results in the release of catecholamines, such as adrenaline (norepinephrine), from the adrenal medulla. The release of adrenaline acts directly on the cardiovascular system triggering peripheral responses, such as an increase in heart rate, respiration and blood pressure (McEwen, 2004). Stress also leads to the release of norepinephrine in the central nervous system via activation of the locus coeruleus, which projects widely to multiple brain regions (Benarroch, 2009). In addition, stress engages the HPA axis by stimulating the release of corticotropin-releasing hormone (CRH) from the paraventricular nucleus of the hypothalamus, which causes the pituitary gland to secrete adrenocorticotropic hormone (ACTH). ACTH in turn stimulates the synthesis and release of glucocorticoid hormones from the adrenal cortex (Ulrich-Lai and Herman, 2009), which bind to mineralcorticoid receptors (MRs) and GRs located throughout the body and brain. Acute activation of the stress response primarily leads to the binding of glucocorticoids to low affinity GRs rather than high affinity MRs, which are occupied under basal conditions (Joëls et al., 2008).

Converging lines of evidence indicate that acute stress affects auditory fear conditioning by stimulating the local release of norepinephrine and activating GRs in the lateral and basal nuclei (BLA) of the amygdala. The most commonly reported effect of stress on auditory fear conditioning is an enhancement in memory consolidation. For example, acute stress or a single systemic injection of the glucocorticoid hormone corticosterone administered immediately after fear conditioning improves long-term memory (Zorawski and Killcross, 2002; Hui et al., 2004, 2006; Roozendaal et al., 2006b). These enhancing effects of a post-training injection of corticosterone on memory consolidation are blocked by a local infusion of the β-adrenergic receptor antagonist atenolol into the BLA, indicating a necessary role for norepinephrine in the amygdala in this corticosterone-induced response (Roozendaal et al., 2006b). Interestingly, it has been reported that infusions of β-adrenergic receptor antagonists into the BLA do not affect consolidation of a conditioned fear memory when administered in the absence of corticosterone (Roozendaal et al., 2006b; Bush et al., 2010). This indicates that norepinephrine only affects fear memory consolidation when the stress response is activated immediately after learning. Evidence also supports a role for direct activation of GRs in the amygdala on memory consolidation. This is based on the observation that blunting the activity of GRs in the BLA with a viral vector impairs long-term memory without affecting acquisition or post-shock freezing (Rodrigues and Sapolsky, 2009).

Further support for the roles of GR activation and norepinephrine release in the amygdala on stress-induced increases in memory consolidation comes from studies using inhibitory avoidance, a complex BLA-dependent form of associative learning. During inhibitory avoidance training, an animal is placed in a brightly lit compartment, with access to a dark chamber. Entering the dark chamber leads to a foot shock and subsequent avoidance of this area. Latency to enter the dark chamber the next day is used to measure retrieval of the fear memory. Like Pavlovian fear conditioning, which involves CS-US pairings, both the acquisition and consolidation of inhibitory avoidance require the activation of NMDA and AMPA receptors in the BLA (Izquierdo et al., 1997). Studies have revealed that consolidation of inhibitory avoidance is enhanced by a single post-training systemic injection of the GR agonist dexamethasone, effects that are replicated by directly infusing the same GR agonist into the BLA (Quirarte et al., 1997). Furthermore, an intra-BLA infusion of propranolol is sufficient to block both the systemic and local effects of dexamethasone, demonstrating that norepinephrine signaling within the BLA is required for GR-mediated enhancement in memory consolidation (Roozendaal et al., 2002). Activation of the GR is also required for norepinephrine-mediated increases in memory consolidation in the inhibitory avoidance task. This was demonstrated by a study in which the enhancing effects of a post-training intra-BLA infusion of a β-adrenergic receptor agonist on consolidation was blocked by pretreatment with an intra-BLA infusion of the GR-antagonist RU 38486 (Roozendaal et al., 2002). In addition to interacting with norepinephrine signaling within the amygdala, glucocorticoids are also able to influence the release of norepinephrine by binding to GRs in the locus coeruleus (McEwen, 1987). For example, infusion of the GR agonist RU 28362 into the locus coeruleus enhances consolidation of an inhibitory avoidance memory (Roozendaal et al., 1999). This effect is blocked by intra-BLA infusions of the β-adrenergic receptor antagonist atenolol, providing further evidence of a role for norepinephrine signaling in the BLA in stress-induced enhancement of memory consolidation (Ferry et al., 1999).

While the above studies support the idea that the GR and β-adrenergic receptors work in concert to enhance the consolidation of BLA-dependent fear memories, the downstream signaling pathways by which fear memories are strengthened are unknown. Based on the known role of the GluA2 subunit of the AMPA receptor in the consolidation of a BLA-dependent memory (Migues et al., 2010), we hypothesize that stress enhances fear memory consolidation by promoting mobilization of the GluA2 subunit to the synapse in the BLA. Evidence of such a mechanism involving mobilization of GluA2 has been observed in the hippocampus. For example, it has been demonstrated that administration of corticosterone increases surface expression of GluA2 in primary hippocampal cell cultures by activating the GR (Groc et al., 2008; Martin et al., 2009). Like memory consolidation, this effect is dependent on protein synthesis (Martin et al., 2009). Similarly in the intact animal, acute platform stress has been shown to increase synaptic GluA2 levels in the hippocampus, as measured by an increase in the co-localization of GluA2 and PSD-95 (Sebastian et al., 2013). Importantly, stress-induced increases in synaptic expression of GluA2 in the hippocampus have been shown to lead to an improvement in long-term memory in the Morris water maze, a change that requires activation of the GR (Conboy and Sandi, 2010). The finding that stress also increases co-localization of GluA2 and PKMζ indicates that PKMζ may be involved in the persistence of memories acquired under conditions of stress by maintaining elevated levels of GluA2 at the synapse (Sebastian et al., 2013). Although stress-induced changes in the mobilization of GluA2 have not been directly assessed in the amygdala, there is indirect evidence that acute stress within the BLA may affect AMPA receptors in a similar manner. In a study using auditory fear conditioning, animals exposed to acute platform stress immediately after a three-tone retention test demonstrated enhanced freezing to presentations of the tone the next day, indicating that stress enhanced reconsolidation of the fear memory (Maroun et al., 2013). This behavioral effect was associated with stress-induced increases in the density of mushroom spines within the BLA (Maroun et al., 2013). Given the necessary involvement of GluA2 trafficking in spine expansion (Passafaro et al., 2003; Matsuzaki et al., 2004), these results are consistent with effects of acute stress on mobilization of this subunit in the BLA under conditions in which stress enhances reconsolidation of a memory. Additional work using slice electrophysiology has shown that application of corticosterone persistently increases the frequency of miniature excitatory postsynaptic currents (mEPSCs) in principal cells of the BLA, the maintenance of which is dependent on GR activation (Karst et al., 2010). While increases in mEPSC frequency may be attributed to increases in the presynaptic release of glutamate, it can also be the result of an increase in the trafficking of AMPA receptors to release sites. Future studies are needed to test whether corticosterone-mediated increases in glutamatergic transmission involve mobilization of GluA2 to the synapse and whether this is a mechanism by which stress could enhance memory consolidation in the amygdala.

Norepinephrine signaling has also been shown to affect mobilization of AMPA receptors. A role for stress in this process is suggested by the finding that norepinephrine and acute predator stress increase GluA1 trafficking to the synapse via phosphorylation of Ser845 in the hippocampus (Hu et al., 2007). In hippocampal cultures, the surface expression of both GluA1 and GluA2 is enhanced by application of the β-adrenergic receptor agonist isoproterenol (Zhou et al., 2012). Interestingly, a lower dose of the same β-adrenergic receptor agonist did not have this effect unless it was combined with corticosterone (Zhou et al., 2012), consistent with a facilitative effect of glucocorticoids on norepinephrine signaling. Based on these findings in the hippocampus, we hypothesize that NE increases trafficking of GluA2 to the synapse in the BLA in a GR-dependent manner. This idea is supported by behavioral studies mentioned above in which norepinephrine-induced increases in memory consolidation were dependent upon glucocorticoids in the BLA (Roozendaal et al., 2002). Supportive evidence is also found in studies using slice electrophysiology that show activation of the β-adrenergic receptor increases AMPA-mediated current and induces long-term potentiation (LTP) in the LA-BA pathway (Liebmann et al., 2009; Pu et al., 2009). However, contrary to our hypothesis, pre-treatment with corticosterone did not promote this effect and instead suppressed the facilitative effect of β-adrenergic stimulation on LTP (Pu et al., 2009). This discrepancy may be accounted for by the dose of corticosterone used, which was three times higher than the dose used to facilitate the effects of a β-adrenergic receptor agonist in hippocampal cell culture (Zhou et al., 2012). Given the opposing effects that can be elicited by different levels of corticosterone (for review see Joëls, 2006), it is possible that a lower dose of corticosterone would facilitate the effects of norepinephrine on LTP induction. Indeed, experiments using slice electrophysiology demonstrate that low to intermediate levels of corticosterone facilitate primed burst potentiation and LTP, while intermediate to high levels of corticosterone suppress synaptic plasticity (Diamond et al., 1992; Kerr et al., 1994). It is also possible that the order in which corticosterone is applied relative to the activation of β-adrenergic receptors is of functional significance. During activation of the stress response in the intact animal, norepinephrine is released immediately, while corticosterone levels peak at a later time point (Droste et al., 2008; Joëls et al., 2011). However in the study by Pu et al. (2009), corticosterone was applied prior to activation of β-adrenergic receptors. Perhaps corticosterone would have enhanced the effects of norepinephrine if it was administered after activation of the β-adrenergic receptors. Support for this idea is found in studies showing that corticosterone suppresses the effects of norepinephrine on inhibitory avoidance when administered before activation of the norepinephrine system (Borrell et al., 1984), and enhances these effects when administered after activation of the norepinephrine system (Roozendaal et al., 2006a). Therefore, it may be important to consider the relative timing in which these two systems are activated in studies utilizing cell culture and slice electrophysiology.

Additional studies are still needed to test whether norepinephrine signaling in the BLA promotes trafficking of GluA2 to the synapse. Furthermore, the mechanisms by which corticosterone and norepinephrine interact to stimulate mobilization of GluA2 to the synapse are important topics for future research.

## Effects of Chronic Stress on Fear Memory: Potential Mechanisms in the BLA

In contrast to acute stress, which promotes immune function and metabolism, chronic stress has been shown to suppress immune function and negatively affect metabolism, increasing the risk for cardiovascular disease (McEwen, 2004). Interestingly, chronic stress leads to adaptations within the hypothalamic-pituitary-adrenal (HPA) axis, such that the response to subsequent stressors is exaggerated (Herman, 2013). For example, exposure to numerous tail shocks for 3 days followed by 1 week of recovery leads to an augmented corticosterone response to one additional tail shock (Servatius et al., 1994). Chronic stress also sensitizes other components of the stress response, including the noradrenergic system. This has been shown in several studies demonstrating that exposure to chronic cold stress prior to acute immobilization stress or exposure to electric shock further enhances the release of norepinephrine in multiple brain regions, including the hippocampus, prefrontal cortex, and bed nucleus of the stria terminalis (Nisenbaum et al., 1991; Gresch et al., 1994; Pardon et al., 2003). Although the same experiments have not been conducted in the BLA, there is evidence that chronic cold stress does enhance the effects of norepinephrine on BLA excitability (Buffalari and Grace, 2009), indicating that chronic stress may exacerbate responses to a future stressor through norepinephrine signaling in the BLA.

Given the known effects of shocks on activation of the stress response and the necessary use of foot shocks during fear conditioning, sensitization of the stress response by pre-exposure to chronic stress may contribute to the effects of chronic stress on fear learning. Numerous studies using auditory fear conditioning have shown that chronic stress or chronic exposure to glucocorticoids before training, either with or without a period of recovery, enhances fear memory (Conrad et al., 1999, 2004; Chou et al., 2014; Monsey et al., 2014; Suvrathan et al., 2014; Hoffman et al., 2015; Marks et al., 2015). While some studies report a moderate effect of chronic stress on fear acquisition (Conrad et al., 1999, 2004; Hoffman et al., 2014, 2015), other reports show selective effects on memory consolidation; that is, only long-term memory is affected (Chou et al., 2014; Monsey et al., 2014; Suvrathan et al., 2014; Marks et al., 2015). Stress-mediated increases in both processes may work together to improve retention of the fear memory.

Chronic stress-induced enhancement of fear memory has been attributed to morphological and physiological changes within the amygdala. For example, chronic immobilization stress increases dendritic arborization of excitatory (principal and stellate) but not inhibitory neurons within the BLA (Vyas et al., 2002). The same chronic stressor also increases the density of spines on primary and secondary dendrites of principal neurons in the BLA (Mitra et al., 2005; Hill et al., 2013; Suvrathan et al., 2014). Additional studies show that chronic immobilization stress enhances LTP at thalamic inputs to principal neurons in the LA, indicating that chronic stress may affect network excitability in the LA in such a way that it becomes more responsive to future emotionally salient events (Suvrathan et al., 2014). Such changes in excitability, in conjunction with stress-induced sensitization of the stress response (see above) may contribute to the enhancing effects of chronic stress on the acquisition and consolidation of a conditioned fear memory.

An investigation into the synaptic changes leading to stress-induced enhancement of LTP revealed that chronic stress increases the ratio of NMDA receptor to AMPA receptor-EPSCs in the amygdala (Suvrathan et al., 2014). In addition, chronic stress lowers the coefficient of variation of NMDA receptor mediated responses, indicating an increase in the number of synaptic NMDA receptors mediating EPSCs. There is also evidence that chronic stress targets AMPA receptors, as demonstrated by stress-induced increases in GluA1-containing AMPA receptors in LA spines (Hubert et al., 2014; Monsey et al., 2014). We hypothesize that the increase in the available pool of GluA1 leads to more GluA1 trafficking during learning, which may contribute to stress-enhanced memory acquisition. After learning, the replacement of GluA1 by GluA2 would then result in enhanced synaptic GluA2, which may be a mechanism by which chronic stress enhances memory consolidation. Although it has not been established that an increase in synaptic GluA1 would necessarily lead to an increase in synaptic GluA2, our hypothesis is in line with the proposal that GluA1 insertion creates synaptic placeholders that reserve the space for later insertion by GluA2 (Barry and Ziff, 2002; Opazo et al., 2012). Interestingly, it has been shown that chronic stress has no effect on the coefficient of variation of AMPA receptors (Suvrathan et al., 2014), indicating no effect of stress on the number of AMPA receptors contributing to the EPSC. In contrast to our hypothesis, one interpretation of these findings is that chronic stress does not increase the number of AMPA receptors and instead produces silent synapses that are devoid of AMPA receptors (Suvrathan et al., 2014). An alternative interpretation is that stress does increase the number of AMPA receptors, but unlike NMDA receptors, they are not as functionally involved in mediating current, because they are consistently trafficked between the synaptic and extra-synaptic membrane in newly formed synapses. Such changes in AMPA receptor mobilization within nascent spines have been demonstrated using quantum dot imaging to track individual receptors (Groc et al., 2006; Hanse et al., 2009). The possibility remains that during learning, these receptors are incorporated into the synapse, where they pass current and contribute to the acquisition of a conditioned fear memory. Furthermore, in the study by Suvrathan et al. (2014), the observed stress-induced increase in the NMDAR/AMPAR ratio remained below 1, indicating that even though stress led to an increase in NMDA receptor mediated current, it was still less than AMPA receptor mediated current, which may also have been increased. Support for this idea comes from the finding that 4 days of foot shock stress increases AMPA-mediated mEPSCs in the LA (Hubert et al., 2014). Future studies are needed to directly test the role of stress-induced changes in AMPA receptors, NMDA receptors, and cell excitability in the BLA on stress-induced enhancement of memory acquisition and consolidation. It should be noted that all studies described in this section have quantified molecular changes in chronically stressed animals that have not been fear conditioned. Given that fear conditioning alone increases expression of GluA1 as early as 5 min after conditioning (Hong et al., 2013), understanding how chronic stress affects this process requires examining GluA1 in stressed and non-stressed fear conditioned animals shortly after conditioning. Similarly, a relevant time point for evaluating the replacement of GluA1 by GluA2 would be 24 h after conditioning in stressed and non-stressed mice. Understanding how chronic stress might lead to changes in NMDA and/or AMPA receptor function through norepinephrine and glucocorticoid signaling would be an interesting subject for future research.

## Implications for Fear Extinction

The inability to extingush memories associated with an aversive experience is commonly found in stress-related psychiatric disorders, such as posttraumatic stress disorder (PTSD; Milad et al., 2009). As a result, understanding how stress affects extinction has garnered considerable attention in the past decade (Maren and Holmes, 2016). The vast majority of studies investigating the effects of stress on extinction expose animals to chronic stress prior to fear conditioning and report stress-induced increases in conditioned responses the next day (Miracle et al., 2006; Wilber et al., 2011; Chauveau et al., 2012; Hoffman et al., 2014, 2015). Although it is possible that this increase in freezing reflects a stress-induced impairment in extinction, this interpretation is confounded by the well established effects of stress on acquisition and consolidation. In other words, animals that learn the CS-US association better will take longer to learn that the CS no longer predicts the US during extinction training.

The relationship between initial acquisition of the fear memory and later extinction of that memory is particularly important, given the differential role AMPA receptor subunits play in each process in the amygdala. During acquisition, GluA1 subunits are rapidly trafficked to the synaptic membrane, and are later replaced by GluA2 subunits during memory consolidation (Hong et al., 2013). During extinction learning, synaptic GluA2 subunits in the LA are internalized (Kim et al., 2007; Dalton et al., 2008, 2012). This process is also required for retrieval of an extinction memory (Chauveau et al., 2012). The opposing roles of synaptic GluA2 in initial consolidation and subsequent extinction is especially relevant when considering how exposure to stress prior to fear conditioning affects each process. If the hypothesis proposed here is correct, and chronic stress increases the amount of GluA2 incorporated into the synapse, then subsequent removal of GluA2 from the synapse during extinction would be expected to take longer, leading to impairments in extinction learning and retention of the extinction memory. If correct, this would indicate that stress-induced enhancement in the acquisition and/or consolidation of a fear memory renders that memory resistant to extinction. However, it would still not address whether stress directly affects the acquisition or retention of an extinction memory. Such studies would involve fear conditioning animals, allowing the memory to be consolidated, and then exposing them to stress prior to extinction learning or retrieval of the extinction memory. To our knowledge, only one study tested the effects of stress on extinction in this way and found that acute platform stress 2 weeks after extinction training impaired the long-term recall of an extinction memory (Deschaux et al., 2013). Given the known role of the medial prefrontal cortex (mPFC) in the consolidation and long-term retention of extinction memories (Milad and Quirk, 2002; Do-Monte et al., 2015), these results indicate a detrimental effect of stress on this brain region (Radley et al., 2004, 2006). Future studies are needed to investigate the direct effects of stress on the acquisition of extinction. Additional studies are also needed to address how stress affects extinction by modifying the network activity of the amygdala and mPFC.

## Potential Clinical Implications

Several lines of evidence implicate the amygdala in the pathophysiology of numerous psychiatric disorders, including PTSD (Rauch et al., 2000; Shin et al., 2004, 2006) depression (Sheline et al., 2001; Thomas et al., 2001), social phobia (Stein et al., 2002) and specific phobias, such as arachnophobia (Lipka et al., 2011). Given that stress is a risk factor for the development of psychiatric disorders (Jacobs and Nadel, 1985; Breslau et al., 1999; Kendler et al., 1999; Risch et al., 2009; Briere et al., 2016), understanding how stress affects the functioning of the amygdala may provide insight into the etiology and treatment of these disorders. In this article, we have reviewed two components of the acute stress response involved in mediating enhanced consolidation of a BLA-dependent memory: activation of the GR and increases in norepinephrine signaling. Pharmacological interventions targeting these systems may have therapeutic value, particularly when combined with cognitive behavioral therapy (Bowers and Ressler, 2015). For example, pharmacologically enhancing memory consolidation by activating GR and/or norepinephrine receptors could strengthen memories formed during therapy. In support of this idea are studies demonstrating that activation of GRs via ingestion of hydrocortisone prior to exposure therapy improves outcome, as measured by a reduction in symptoms in patients with PTSD (Yehuda et al., 2015), acrophobia (Dominique et al., 2011), social phobia (Soravia et al., 2006) and arachnophobia (Soravia et al., 2006). Similarly, increasing the release of norepinephrine with yohimbine, an α2-adrenergic receptor antagonist, prior to exposure therapy has been shown to be effective in treating social phobia (Smits et al., 2014) and claustrophobia (Powers et al., 2009). Alternatively, blocking memory consolidation by targeting these same components of the stress response could be used to disrupt the formation of a traumatic memory. Studies utilizing this approach provided propranolol shortly after exposure to a traumatic event and found that it decreased the later development of PTSD (Vaiva et al., 2003), an effect that was dependent on high drug adherence (Hoge et al., 2012). However, propranolol did not affect PTSD symptoms when it was administered to patients up to 48 h after physical injury (Stein et al., 2007), a result that may be accounted for by the narrow time window during which memories are consolidated. It is possible that the component of consolidation that is sensitive norepinephrine does not last 48 h and targeting consolidation with propranolol is only effective if it is given closer to the time of trauma. This possibility highlights the importance of tracking the time between trauma exposure and propranolol administration. Perhaps this is why one study that did not monitor the time between drug administration and trauma found no effect of propranolol on the development of PTSD (McGhee et al., 2009). It should also be noted that the vast majority of studies targeting consolidation with propranolol have been conducted in adults and propranolol may not be therapeutically effective in children (Nugent et al., 2010). Another strategy for disrupting traumatic memories has been to interfere with memory reconsolidation, in which case patients are treated after a traumatic memory has already been consolidated. Administration of propranolol during or immediately after retrieval of the fear memory has been shown to impair reconsolidation (Kindt et al., 2009; Soeter and Kindt, 2015), leading to a decrease in PTSD symptoms (Brunet et al., 2008, 2011; but see Wood et al., 2015). Although it is currently unknown whether blocking GR activation immediately after trauma or during retrieval would be similarly beneficial, studies conducted in rodents indicate that this may be a promising avenue of future research (Tronel and Alberini, 2007). Together these studies indicate that pharmacologically targeting GR activation or norepinephrine signaling to either enhance memories formed during therapy or impair memories formed during a traumatic event are promising treatment strategies.

## Conclusions and Future Directions

Understanding how stress affects amygdala-dependent memories is an area of ongoing research. Converging lines of evidence indicate that acute stress enhances consolidation of a fear memory by activating GRs and stimulating the local release of norepinephrine in the amygdala. However, the downstream signaling pathways have not been identified. Based on the known role of GluA2-containing AMPA receptors in the BLA in memory consolidation, we hypothesize that stress enhances consolidation by promoting mobilization of the GluA2 subunit to the synapse. In contrast to the acute effects of stress, chronic stress has been shown to enhance both the acquisition and consolidation of a fear memory. Based on evidence indicating that chronic stress increases the available pool of GluA1 in the BLA, we hypothesize that there are consequent increases in GluA1 trafficking during learning that contribute to the effects of chronic stress on acquisition. After learning, the replacement of high levels of synaptic GluA1 with GluA2 may be a mechanism by which chronic stress enhances memory consolidation. Investigating how stress-induced changes in glucocorticoid and norepinephrine signaling might affect emotional learning by increasing the trafficking of AMPA receptors and enhancing amygdala excitability is an interesting area for future research.

## Author Contributions

AVA provided the hypotheses and wrote the manuscript. NSB and PAS wrote and edited the manuscript.

## Funding

This project was supported in part by the RCMI grant number RR003037 from the National Center for Research Resources (NCRR); National Institutes of Health (NIH) 5R24DA012136-13 and 1R21 MH109779-01 to PAS and by the National Institute on Minority Health and Health Disparities of the NIH under award number G12MD007599 to NSB.

## Conflict of Interest Statement

The authors declare that the research was conducted in the absence of any commercial or financial relationships that could be construed as a potential conflict of interest. The reviewer AK and handling Editor declared their shared affiliation, and the handling Editor states that the process nevertheless met the standards of a fair and objective review.
